# Monitoring the Wobbe Index of Natural Gas Using Fiber-Enhanced Raman Spectroscopy

**DOI:** 10.3390/s17122714

**Published:** 2017-11-24

**Authors:** Vincenz Sandfort, Barbara M. Trabold, Amir Abdolvand, Carsten Bolwien, Philip St. J. Russell, Jürgen Wöllenstein, Stefan Palzer

**Affiliations:** 1 Laboratory for Gas Sensors, Department of Microsystems Engineering–IMTEK, University of Freiburg, Georges-Köhler-Allee 102, 79110 Freiburg, Germany; vincenz.sandfort@imtek.uni-freiburg.de; 2 Max Planck Institute for the Science of Light, Staudtstraße 2, 91058 Erlangen, Germany; barbara.trabold@mpl.mpg.de (B.M.T.); aabdolvand@ntu.edu.sg (A.A.); philip.russell@mpl.mpg.de (P.S.J.R.); 3 School of Electrical and Electronic Engineering, Nanyang Technological University, 50 Nanyang Ave, Singapore 639798, Singapore; 4 Fraunhofer Institute for Physical Measurement Techniques IPM, Heidenhofstraße 8, 79110 Freiburg, Germany; Carsten.Bolwien@ipm.fraunhofer.de (C.B.); Juergen.Woellenstein@ipm.fraunhofer.de (J.W.); 5 Department of Computer Science, Universidad Autónoma de Madrid, Francisco Tomás y Valiente 11, 28049 Madrid, Spain

**Keywords:** Raman spectroscopy, wobbe index, photonic crystal fiber, kagomé, fiber enhanced, natural gas

## Abstract

The fast and reliable analysis of the natural gas composition requires the simultaneous quantification of numerous gaseous components. To this end, fiber-enhanced Raman spectroscopy is a powerful tool to detect most components in a single measurement using a single laser source. However, practical issues such as detection limit, gas exchange time and background Raman signals from the fiber material still pose obstacles to utilizing the scheme in real-world settings. This paper compares the performance of two types of hollow-core photonic crystal fiber (PCF), namely photonic bandgap PCF and kagomé-style PCF, and assesses their potential for online determination of the Wobbe index. In contrast to bandgap PCF, kagomé-PCF allows for reliable detection of Raman-scattered photons even below 1200 cm^−1^, which in turn enables fast and comprehensive assessment of the natural gas quality of arbitrary mixtures.

## 1. Introduction

The analysis of complex gas matrices at reasonable cost is an often required but seldom accomplished task and applications for such a technology range from air quality management to process monitoring as well as safety and security applications. One of the world’s biggest markets for multi-gas analysis equipment is in the natural gas sector, where fast and reliable determination of the Wobbe index [1,2,3,4] is much sought after. The Wobbe index is used as a measure for the interchangeability of fuel gases and indicates their combustion energy.

Currently, Fourier transform infrared spectroscopy (FTIR [5,6,7,8]), gas chromatography (GC [9,10]) and mass spectrometry (in particular coupled with GC: GC-MS [11,12,13]) are the most common laboratory-based tools whenever the constituents of the gas mixture are unknown but their identification is required. Other, more cost-effective technologies, such as e.g., metal oxide based gas sensors [14] or standalone flame ionization detectors [15], show a high degree of sensitivity to many gases, but do not allow specific detection and identification of individual gas components. Among the optical methods, infrared absorption spectroscopy tools such as tunable diode laser spectroscopy (TDLAS) or non-dispersive infrared spectroscopy (NDIR) allow selective and sensitive detection of individual components [16,17,18,19,20]. However, they are unsuitable in terms of total cost in scenarios with many different gases because many laser sources are needed to perform TDLAS on the distinct absorption lines or many spectral filters in case of NDIR.

Raman spectroscopy offers the opportunity to simultaneously measure gas mixtures [21,22,23,24] using a single light source. It is based on the Raman effect, experimentally first observed by Krishnan and Raman in 1928 [25], and relies on the change of energy of photons that are scattered during the interaction with matter. While it is well known and widespread for analyzing solids in crystallography and liquids in chemistry [26], it is less often used in commercial products for gas analysis as compared to absorption spectroscopy techniques. However, important applications include the analysis of reacting flows [25] or atmospheric temperature determination [27,28,29,30]. This is due to the typically small Raman scattering cross-sections $\sigma_{Raman}\left(\lambda_{0}\right)\sim {\left(\frac{1}{\lambda_{0}}\right)}^{4}$ ($\lambda_{0}$ is the excitation wavelength), which are several orders of magnitude below the absorption cross-section $\sigma_{Abs}$ of infrared active lines used in TDLAS [31]. Compared to solids or liquids the Raman scattering rate in gas is typically three orders of magnitude lower due to the lower density. According to [32,33,34] the detected Raman intensity $I_{Raman}$ depends on the excitation laser intensity $I_{Laser}$, the number of molecules N in the illuminated volume V and the effective interaction length *l_eff_* :

(1) $$I_{Raman}=\varepsilon \cdot I_{Laser}\cdot \frac{N}{V}\cdot l_{eff}\cdot \sigma_{Raman}$$

for a Raman apparatus with total detection efficiency ε. The obvious possibilities to increase *I_Raman_* are increasing the particle number N, increasing the excitation intensity $I_{Laser}$, or using shorter excitation wavelengths. For this reason several strategies are being pursued for enhancing the scattered light intensity when probing gases. Enhancing the signal may be achieved using non-linear techniques such as coherent anti-Stokes Raman spectroscopy (CARS [35,36,37]) or stimulated Raman spectroscopy (SRS [37,38,39]). While these techniques require at least two laser sources whose frequency difference may be tuned, the Raman signal may be amplified by several orders of magnitude as compared to spontaneous Raman scattering. However, the more complicated optical setups and limitations imposed by the frequency range that may be tuned limit the range of applications outside the laboratory. On the other hand, non-coherent Raman spectroscopy methods do require only one laser source, albeit at a lower scattering intensity. Commercial products for probing gas samples are on the market [40,41] and several strategies are being investigated for enhancing the scattered light intensity when probing gases using a single laser source to improve signal strength and widen the range of possible applications. To this end, multipass cells [42,43,44,45,46,47] and capillary fibers [48,49] allow for amplifying the Raman signals. One prominent possibility relies on using optical resonators to enhance the light intensity available for scattering (CERS-cavity enhanced Raman spectroscopy) [50,51]. Another approach uses hollow-core photonic crystal fiber (HC-PCF) to enhance the number of probed molecules by increasing the interaction length with the gas and additionally guiding the scattered photons (FERS-fiber enhanced Raman spectroscopy) [21,22,52,53]. In this way a photonic bandgap (PBG) HC-PCF has been used to increase the Raman scattering signal by several orders of magnitude [54] depending on the type and length of the fiber. To estimate the enhancement factor of a HC-PCF as compared to a focused laser beam one may compare the effective interaction lengths *l_eff_*. In case of a free-space beam the collecting optics can only deliver a certain part of the scattered signal to the spectrometer, limiting *l_eff_* to roughly twice the Rayleigh length. For a free running laser beam at 532 nm with a beam diameter of 4 µm and a Rayleigh range *z_R_* of 23.6 µm the effective interaction length is thus $l_{eff}=2\cdot z_{R}=2\cdot \pi W_{0}^{2}/\lambda =47.2\;\mu m$. Comparing this to the interaction length of a HC-PCF 1 m in length yields an amplification factor of approximately 21,000. However, a direct comparison between Raman systems with and without [55] fibers is difficult because the total detection efficiency is dependent on the quality of the Raman light collection optics, which is not standardized.

In the past the enhancement capabilities of FERS have been employed to demonstrate the feasibility of using Raman spectroscopy in demanding applications such as breath gas analysis [52], isotope selective greenhouse gas detection [56], and natural and biogas analysis. Among the various possible applications, one of the biggest markets is the fast and reliable analysis of natural gas compositions [57,58,59]. While in prior work the use of a cavity-enhanced setup to detect some natural gas components such as methane, propane, ethane, and hydrogen sulfide was reported [51], less effort has been devoted to assessing its suitability for online monitoring of natural gas. However, natural gas supply and transport utilities are keen to determine the so-called Wobbe index [1,3,4]. The Wobbe index is a measure for the heating value of a gas composition and is the most-frequently used indicator for the quality of natural gas and the interchangeability of different gas types [2,60,61]. Determination of the Wobbe index requires identification and quantification of the hydrocarbon components, which typically feature Raman shifts between 800 cm^−1^ and 3100 cm^−1^, and other further constituents like CO_2_ and N_2_. Using Raman spectroscopy may offer a convenient, fast, and reliable alternative overcoming the drawbacks of established technologies like FTIR, TDLAS, and NDIR. However, some important components of natural gas mixtures, e.g., ethane, propane and *n*-butane, feature Raman peaks in the region below 1200 cm^−1^, which are covered by the background signal from the silica cladding. The glass background signal makes it difficult to record signals from the gas, even though out the light emitted from the cladding or, alternatively, analyzing peaks in the CH-stretching region around 3000 cm^−1^, may improve the situation. But due to the many CH-stretching modes of many hydrocarbons in this region this requires a very fine resolution of the measurement to resolve the different modes of different gases.

Here, we demonstrate for the first time the use of a kagomé fiber for FERS analysis of natural gas as a much simpler alternative to suppress the glass background, simply because the light fraction in the glass is predicted to be about 10 dB lower than in PBG-PCF. On that basis, we investigate the possibility of using FERS as a fast, reliable and cost efficient method for determining the Wobbe index. The performance of kagomé-PCF and PBG-PCF is compared in terms of acquisition time and signal to noise level when analyzing a natural gas composition. To evaluate the practical issue of measurement frequency when using HC-PCF, the gas exchange times are determined for both fiber types and an experimental approach to minimize the effects of dead volumes is presented. Other techniques to reduce the evacuation times of photonic crystal fibers include micro-channel machining [62,63] might be used additionally. While kagomé-PCF is known for a low Raman signal background [64], the typically larger core diameter results in a weaker signal strength. The fiber length is optimized, which means making a compromise between short gas exchange times and high Raman signal levels.

## 2. Setup and Methods 

**Figure 1:**
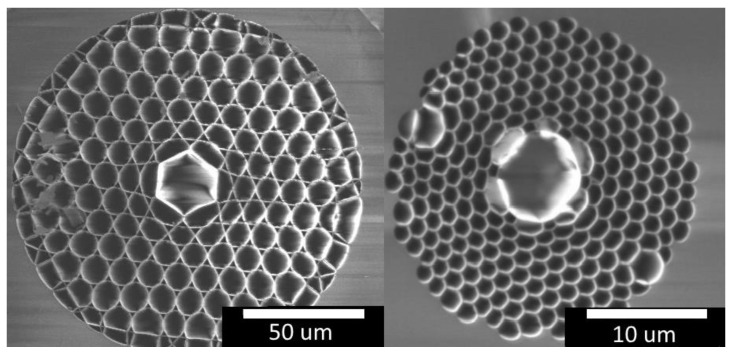
Scanning electron microscopy images of the kagomé-PCF (**left**) and a photonic bandgap PCF (**right**). The core diameters are 12 µm and 2.25 µm, respectively. Please note the 5-fold difference in magnification.

A system for Raman spectroscopy [54] has been adapted for gas measurements in HC-PCF. Confinement of gas and guidance of light are achieved in hollow-core PBG-PCF and kagomé-PCF, respectively. Both are expected to exhibit different behavior in terms of gas exchange rates, background signals and enhancement factors. The PBG-PCF used is a “HC-580” fiber from NKT Photonics (Birkerød, Denmark), with a core radius of 2.25 µm. The alternatively available “HC-532” has a lower attenuation at 532 nm, but for Raman measurements up to 3500 cm^−1^ the fiber has to deliver a little attenuation of the Stoke photons with wavelengths up to 632 nm. This is why the “HC-580” is more suitable for this specific task. Its performance is compared to a custom-made kagomé-PCF [64,65] with a core radius of 12 µm. Varying fiber lengths between 5 cm and 80 cm are used. Figure 1 shows scanning electron micrographs (SEMs) of the in-coupling facet for both fiber types.

**Figure 2:**
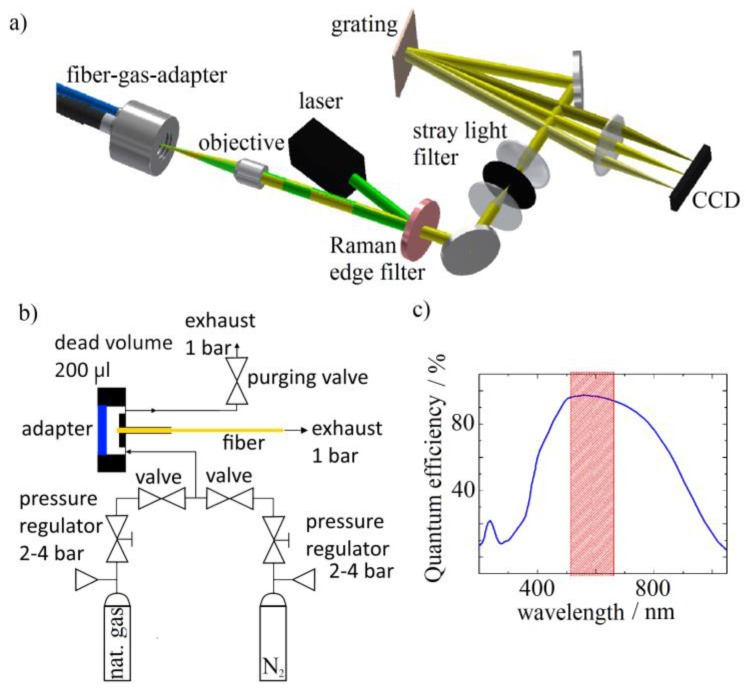
(**a**) Schematic drawing of the setup. The excitation light is depicted in green and the Raman-scattered in yellow; (**b**) Plumbing diagram for the gas delivering system with the hollow fiber (yellow); (**c**) Quantum efficiency of the Andor iDus401-BVF camera. The red-shaded region marks the spectral range used.

The experimental setup is depicted in Figure 2a. A frequency doubled Nd: YAG laser (COMPASS 215M50) at 532 nm is used as light source and the light is coupled into the HC-PCFs using a Raman edge filter (Semrock SEM-LP03-532RE-25) and a microscope objective. The input fiber facet is also used as output port for the backward scattered Raman photons. In this way, the objective collimates the Raman-scattered light and the edge filter disposes of the excitation wavelength. To enable simultaneous coupling of light and gas into the fiber, it is mounted in a gas-tight adapter (Figure 2b) that can withstand up to 14 bar. The dead volume of 200 μL may be rinsed through a purging valve, which opens up the dead volume to air. Optical access is gained through a 0.5 mm thick sapphire window. Two connectors attached to the adapter provide gas-tight interfaces to the optical fiber and a gas delivery system, respectively. The latter is derived from a more sophisticated setup for control of gas compositions in laboratory environments [66]. The fiber is connected via an SMA905-thread and the gas delivery system via an M3-thread for gas connectors. The other fiber end facet is left open to a gas extraction system operating just below ambient pressure.

The back-scattered light is then directed onto a spectrometer, with a 50-µm-pinhole filtering out parts of the stray light. A diffraction grating with 1800 lines/mm is used to spatially separate the Raman-scattered photons according to their wavelengths. The spectrum is imaged onto a CCD camera (iDus401-BVF, Andor Technology, Belfast, UK), which records the intensity of scattered light around the pump beam frequency ${\bar{\nu }}_{0}\;\approx$ 18,800 cm^−1^ in the range from ${\bar{\nu }}_{0}$ = −100 cm^−1^ to ${\bar{\nu }}_{0}$ = +3500 cm^−1^ using the full length of the CCD array of (1024 × 127) pixels with a pixel size of (26 × 26) µm^²^ at a distance of 107.5 mm between grating and camera resulting in a spectral resolution of ~8 cm^−1^. The camera is set to full vertical binning mode, 16.25 µs vertical pixel shift time, 50 kHz horizontal pixel readout rate and 1.0× pre-amp gain. With these camera settings, every electron is converted to 4.7 A/D-counts on average. For every pixel, the quantum efficiency *QE(λ)* of the camera for the corresponding wavelength is given in Figure 2. The detected Raman scattering rate *R_Raman_* provided in the graphs below is calculated using the expression:

(2) $$R_{Raman}=\frac{N_{ph}}{t_{Int}}=\frac{N_{e}}{QE\left(\lambda \right)\cdot t_{Int}}=\frac{C}{4.7\cdot QE\left(\lambda \right)\cdot t_{Int}}$$

where *N_ph_* is the number of incoming photons, *N_e_* is the number of excited electrons, *t_Int_* is the integration time and *C* is the number of A/D counts. The total scattering rate for each gas species is determined by integrating the values of all corresponding pixels of the respective Raman peak. Coupling into the fiber was achieved by a 3-dimensional microscope stage. All measurements are performed at an ambient temperature of 23 °C and an outlet pressure of ~1 bar at the exhaust. The natural gas composition used in the experiments is a sample blend purchased from Air Liquide (Paris, France) and is composed of the most prominent, typically occurring natural gas constituents according to the list in Table 1. To change the gas composition inside the fiber the gas mixture is fed in via the gas connection at an absolute pressure of up to 4 bar. To evaluate and optimize the gas exchange rate caused by the dead volume, experiments with and without using the purging valve were performed.

With the setup in Figure 2, the full Raman spectrum can be analyzed in a single measurement, enabling determination of the gas composition. This makes the technique suitable for on-line determination of the Wobbe index. In general, inferior and superior limits are given for the Wobbe index, namely the inferior Wobbe index $W_{i}$ and the superior Wobbe index $W_{S}$. They can be calculated from the lower heating value $H_{i}$ and the gross heating value $H_{s}$ as [67]:

(3a) Wi=Hiρ/ρ0

(3b) WS=HSρ/ρ0

where ρ is the gas density and $\rho_{0}$ the density of dry air. The lower and gross heating values for the contained gases, as well as their individual densities for 100% purity, are listed in Table 2. The Wobbe index of a gas mixture is the concentration-weighted mean value of the individual Wobbe indices. Here, the relative concentrations of the individual components are deduced from the measured Raman spectra, which allows real-time calculation of the Wobbe index.

## 3. Results

**Figure 3:**
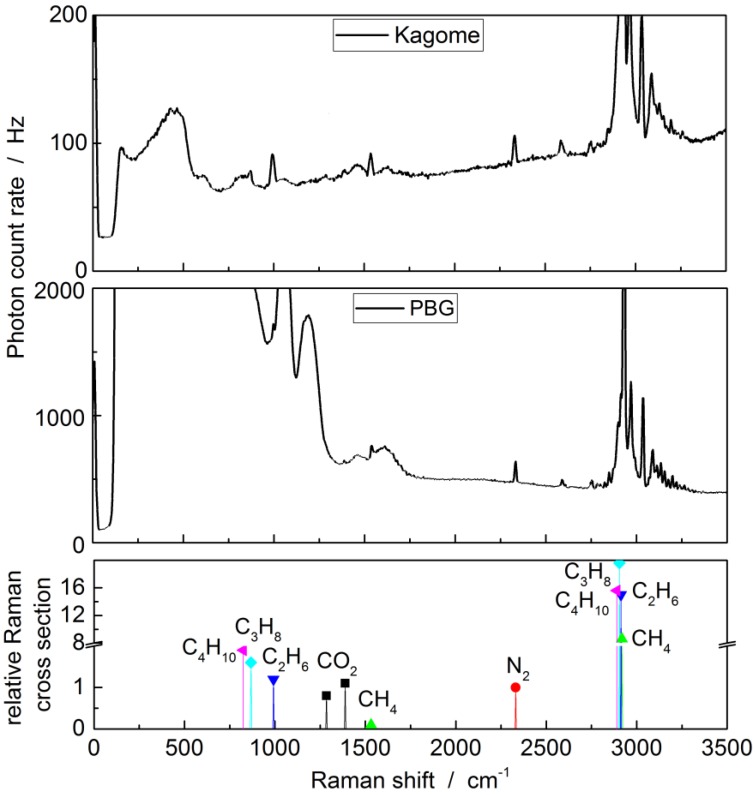
Spectra of the Raman scattering of natural gas for an input laser power of 27 mW. Displayed are scattered photons per second over the Raman shift in wavenumbers, i.e., inverse centimeters. Note the different vertical scales for the upper and middle panel. The large glass background for the PBG fiber can be seen from approximately 100 cm^−1^ to 1700 cm^−1^. The lower panel displays the position of the different Raman peaks of the gas components. The relative Raman scattering cross-section for each molecule is normalized to that of nitrogen [37,75,76,77].

Filling both fiber types with the natural gas mixture at an input pressure of 2 bar and a length of 80 cm results in the spectra shown in Figure 3. The light coupled into each fiber was held constant for all measurements at 27 mW for the PBG-PCF and 28 mW for the kagomé-PCF, respectively. Notably, the PBG-PCF generates a strong background signal, ranging from approximately 0 to 1700 cm^−1^, even partly saturating the camera at an integration time of 0.5 s. This is due to the non-negligible portion of light propagating in the SiO_2_ cladding material [71,72,73], which, as a solid, features orders of magnitude higher Raman gain as compared to a gas. In the kagomé-PCF, however, the glass background is strongly suppressed because the electric field penetrating the glass is typically around 10 dB lower as compared to PBG-PCF [74].

The corresponding molecular spectra obtained using a kagomé-PCF show a much reduced scattering rate, i.e., the integrated nitrogen peak is about 4 times lower than in the PBG-PCF. This is due to the larger core diameter of the kagomé-PCF, which results in a smaller acceptance angle for the scattered light to be guided in the core. Nevertheless, the reduced background scattering from glass allows the detection of natural gas components including butane and propane below 1700 cm^−1^, which could not be detected using the PBG fiber.

**Figure 4:**
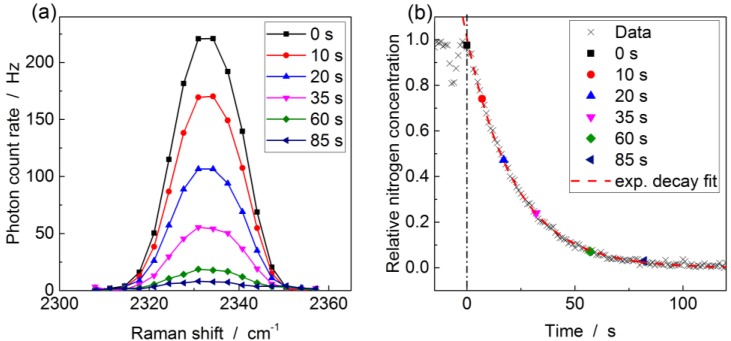
Procedure for evaluating the total nitrogen Raman scattering rate, illustrated using a 0.8 m length of kagomé-PCF at an absolute input pressure of 2 bar. (**a**) Nitrogen peak around 2331 cm^−1^ at various times during the gas exchange; (**b**) Transient plot of the integrated Raman scattering rate during gas exchange, which starts at *t* = 0 s. An exponential fit yields the *t*_90_ time.

In order to evaluate the practical suitability and attainable measurement speed, the gas exchange times and the signal strength for different lengths and input pressures are measured. Therefore, pure nitrogen gas is substituted by the natural gas mixture and the Raman peak of N_2_ recorded during the exchange. A sample measurement for 2 bar input pressure with closed purging valve is shown in Figure 4a. The nitrogen Raman peak is localized around 2331 cm^−1^, which results in a peak extending from 2311 cm^−1^ to 2355 cm^−1^ detected on the camera, i.e., 11 pixels when using the configuration of the spectrometer setup above. To evaluate the total N_2_ scattering rate the background signal of the camera is subtracted before integrating over all involved pixels, to obtain the total Raman scattering rate displayed in Figure 4b. The *t*_90_ time, i.e., the it takes to reach 90% of the new steady-state reading, is deduced by applying an exponential decay fit (red dashed curve), resulting in 47 s for the measurement in Figure 4b.

**Figure 5:**
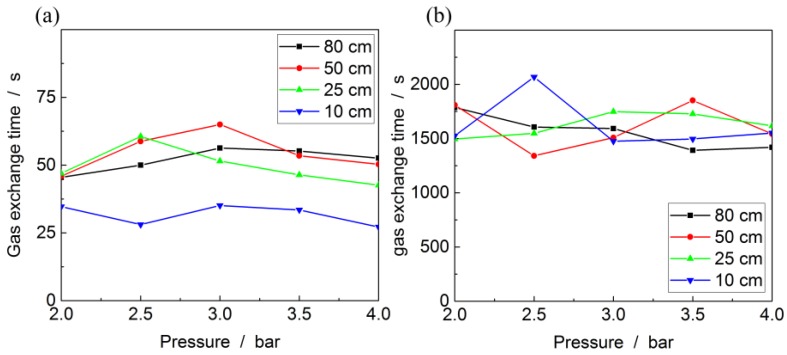
Gas exchange times of (**a**) kagomé-PCF and (**b**) PBG-PCF at lengths of 0.1 m, 0.25 m, 0.5 m and 0.8 m. Please note the different scales on the *y*-axis.

To determine the gas exchange times for different fiber lengths and input pressures, Raman spectra are recorded at a frequency of 1 Hz for the kagomé-PCF and 2 Hz for the PBG-PCF. The resulting t_90_ times are shown in Figure 5. There is a negligible correlation between the input pressure and the gas exchange time and also the fiber length does not appear to have a major influence. When entering the fiber, the gas velocity increases due to the conservation of mass principle and at the same time the Venturi effect lowers the pressure behind the fiber. At a fixed input pressure the mass flow will not increase beyond a limiting condition, i.e., the so-called critical valve or choked flow effect [78], which ultimately results in a constant flow velocity inside the fiber. The small fiber core diameters and the applied pressure differences of Δp≥1 bar at a minimum pressure of 1 bar lead to a Knudsen number $K_{n}\ll 1$ and laminar flow [79], in contrast to free molecular flow that appears for Knudsen numbers $K_{n}\gg 1$. For photonic crystal fibers operated in varying regimes these flow regions are discussed in detail in [80,81].

The mean gas exchange time here is 49.1 s with a standard deviation of 16.3 s for the kagomé-PCF and 1466 s with a standard deviation of 332 s for the PBG-PCF. That means gas flow into the kagomé-PCF is about 30 times faster than into the PBG-PCF. As the kagomé-PCF’s core area is 28 times larger, the diameter of the fiber core is identified as the decisive factor, which is in line with expectations from the critical valve effect. The fibers used in here feature core holes much larger than the surrounding cladding holes, which is why the gas exchange will predominantly happen where the gas is detected as well. While we do not anticipate any interference by slower gas exchange rates in the cladding, should such effects occur they may be eliminated by selectively closing the cladding holes of the input facet for gas [82].

Because the gas in the dead volume has to be exchanged via the HC-PCF, the measured gas exchange times are on the order of minutes, which makes the design of small dead volumes a priority in this type of setup. Another method consists of replacing the gas in the dead volume with the help of the purging valve such that only the gas volume actually filling the fiber needs to flow through the fiber. To this end, experiments have been performed employing PBG-PCF with lengths of 15 cm and 27 cm. The experimental protocol is altered so as to allow purging of the dead volume for five seconds prior to starting data capture of the Raman signal. Because the purge valve has a diameter of 1 mm the dead volume is purged mostly through the purging valve, while the gas composition inside the hollow fiber remains constant.

**Figure 6:**
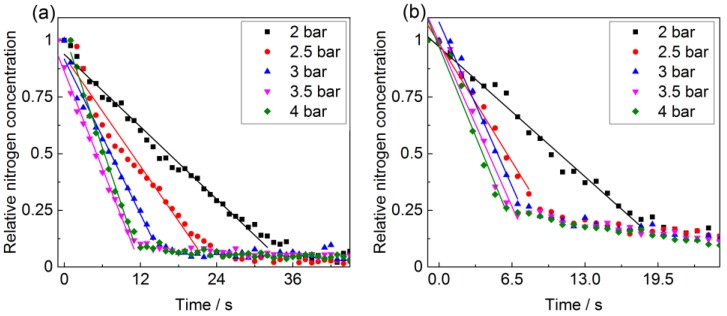
Normalized nitrogen scattering rate during exchange with natural gas from PBG-PCF with lengths of (**a**) 27 cm and (**b**) 15 cm. The purge valve is used to quickly change the gas in the dead volume in front of the fiber. Once it is closed, the measurement starts. Notably, the decrease in scattering rate is now linear during the gas exchange process.

**Figure 7:**
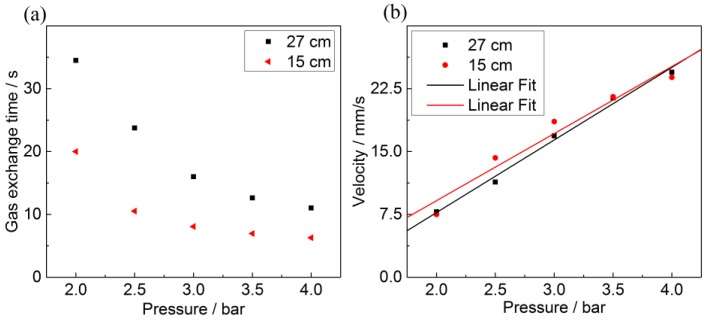
(**a**) Gas exchange time of the PBG fiber as a function of the input pressure when employing the purging valve; (**b**) Gas exchange velocity for various pressures and fiber lengths of 15 cm and 27 cm, respectively.

In Figure 6 the time-dependent Raman signal for nitrogen is shown after the purge valve has been closed. The nitrogen molecules inside the fiber are now replaced by the natural gas mixture within seconds. Comparing the temporal behavior in the Figure 4 and Figure 6 reveals important differences between the two approaches. Gas exchange via the dead volume (cf. Figure 4) results in an exponential decay associated with the mixing process of both gas samples in the dead volume. If the dead volume is rinsed first, the decrease in scattering rate is linear with time. Employing a linear fit to the N_2_ Raman scattering rate in Figure 6, the slope *b* can be determined and used to calculate the gas exchange time *t*_90_ = −0.9/*b* shown in Figure 7a. From this, the gas flow velocity *v*_ge_ is determined and plotted in Figure 7b. The exchange time now linearly depends on the pressure and is reduced from 1466 s on average to tens of seconds, i.e., by more than two orders of magnitude for the PBG-PCF.

The results in Figure 7 correspond well with basic considerations where the natural gas pushes the nitrogen out of the fiber without mixing. Assuming a laminar flow inside the hollow fiber as well as the validity of Hagen-Poiseuille’s equation one would expect a gas flow velocity according to [79]:

(4) $$v_{m}\left(\Delta P\right)=\;\frac{1}{4\eta }\cdot \;\frac{R^{2}}{l}\cdot \Delta P=G_{v}\cdot \Delta P,$$

where *R* is the fiber core radius, *l* the fiber length, η the viscosity of the gas filling, i.e., $\eta_{N2}$ = 17.7 µPa s for N_2_ at room temperature, and ΔP=Pi−Po the pressure difference between input *Pi* and output pressure *Po*. The velocity increase per bar is labeled *Gv*.

*Gv* can be determined experimentally from the slope of the linear fits in Figure 7b. However, while this yields the correct order of magnitude for the gas exchange time, the fiber length appears to have no bearing on the gas velocity inside the fiber. This is also due to the choked valve effect, which limits the mass flow. In fact, applying a linear fit to the gas exchange velocity as a function of the input pressure results in slopes for both lengths that are equal within the margin of error. Table 3 summarizes these findings. Using Equation (4) is then suitably only to gives an estimation of the correct order of magnitude for *Gv*. This model is also applicable for the kagomé-PCF, which should result in even faster gas exchanged times (c.f. Equation (4)).

**Figure 8:**
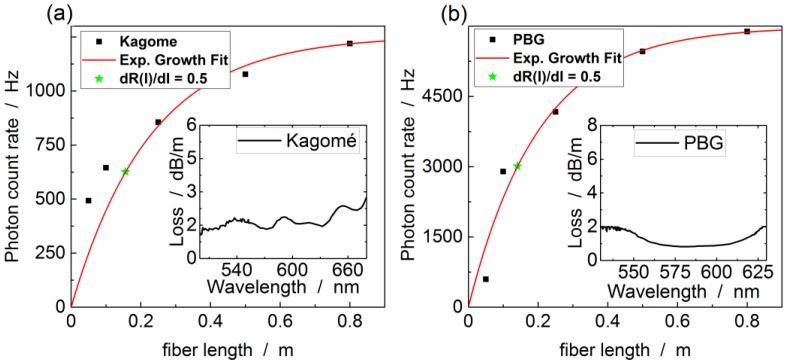
Integrated nitrogen signal plotted over the fiber length at 2 bar pressure for (**a**) kagomé-PCF and (**b**) PBG-PCF. A logarithmic fit is applied to take account of the two-way-attenuation in the fiber. Insets show the loss of the fundamental mode specified by the manufacturer.

From Figure 7a and Equation (4), it is clear that a short gas exchange time requires a short fiber length, which conflicts with the need for a long fiber to maximize the Raman signal. In the following, the influence of the fiber length on the detected scattering rate is investigated so as to determine the ideal fiber length. The light coupled into each fiber length was held constant at 27 mW for the PBG-PCF and 28 mW for the kagomé-PCF. The measured Raman scattering signals are shown in Figure 8. The nonlinear dependence of the Raman scattering rate on fiber length is mainly caused by fiber loss, resulting in two separate effects. Firstly, the exponential decrease in pump intensity along the fiber reduces the number of generated Raman photons. Secondly, the Raman scattered photons are attenuated on the way back to the output port. These losses are further increased by coupling of Raman scattered photons into higher order guided modes that have higher losses than the fundamental mode. Pump and Stokes attenuation counteract the linear increase of the scattered photon number with the fiber length (see Equation (1)). For this reason the scattering rate saturates with increasing fiber length.

Based on the theoretical considerations above, the data can be fitted with the following model (red curve in Figure 8):

(5) $$R_{Raman}\left(L\right)\sim \int_{0}^{L}J_{0}\cdot e^{-\left(\alpha_{P}+\alpha_{S}\right)\cdot l}\;dl=\frac{J_{0}}{\alpha_{P}+\alpha_{S}\;}\;\left(1-e^{-\left(\alpha_{P}+\alpha_{S}\right)\cdot L}\right),$$

where *L* is the fiber length, $\alpha_{P}$ and $\alpha_{S}$ the pump and Stokes attenuation coefficients, respectively, and $J_{0}=I_{0}/m$ is initial laser power per meter of the fiber. The resulting fit parameters are summarized in Table 4. The value for $\alpha_{S}+\alpha_{P}$ in Table 4 is considerably higher than predicted by the loss curves in the insets of Figure 8. This happens because higher-order guided modes losses range from few dB/m to above 100 dB/m loss [83].

To quantify the performance of both fibers as a function of their lengths, the differential Raman scattering rate gain $R^{\prime }\left(l\right)=dR_{Raman}\left(l\right)/dl$ has been calculated, i.e., the slope of the curves in Figure 8. The ideal fiber length *l_ideal_* has been defined as $R^{\prime }\left(l_{ideal}\right)=0.5\;R^{\prime }\left(0\right)$ and is indicated by the green marks in Figure 8. This leads to ideal FERS fiber lengths of 14.1 cm for the PBG-PCF and 15.6 cm for the kagomé-PCF.

Using the Raman scattering rates from Figure 8 for the ideal fiber length, the limit of detection (*LOD*) for both fiber types has been determined for the most prominent components of natural gas. The *LOD* is defined as [84]:

(6) $$LOD=3\cdot \frac{c}{SNR},$$

where c is the gas concentration and SNR is the measured signal-to-noise ratio. Noise is mainly generated by the standard deviation of the signal and the background noise of the camera caused by stray light and random detection events. The signal-to-noise ratio is calculated from the signal amplitude S and the square root of the the standard deviation of the error σ [85]: SNR=Sσ. Since the photon scattering rate is an integral over a multiple of camera pixels, the derived background noise is the sum of the used pixels for every band. The resulting excitation-intensity dependent *LOD*s are listed in Table 5 in ppm at 2 bar pressure and 1 s integration time, together with their associated Raman shifts in wavenumbers and the relative Raman scattering cross sections at that shift. The Raman bands used for the calculation of the hydrocarbon values are in the region from 800 cm^−1^ to 1600 cm^−1^, due to the mentioned band overlaps in the C-H stretching region around 3000 cm^−1^. With a high resolution spectrometer and chemometrics the upper bands may be used as well albeit at the cost of using considerable more complex data analysis methods.

**Figure 9:**
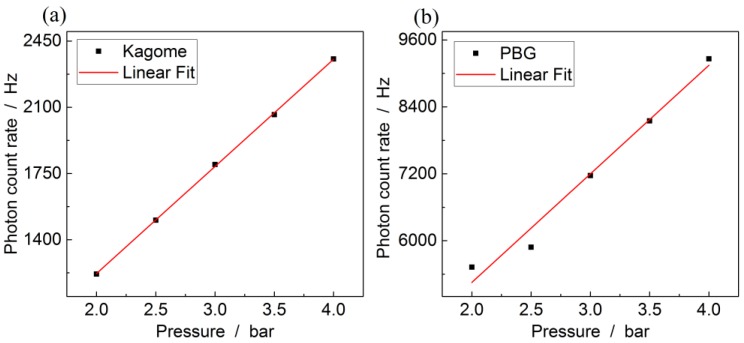
Integrated nitrogen signal plotted over the pressure for (**a**) kagomé-PCF and (**b**) PBG-PCF. The red curve shows a linear regression.

Increasing the input pressure leads to a linear increase of the number of molecules inside any given fiber. The pressure-dependent scattering rate of nitrogen is plotted in Figure 9 for both fiber types at a length of 0.8 m. Both show a linear increase with the number of molecules and according to Equation (1) also with the laser power. For example, the LOD for nitrogen at the currently used laser power and an input pressure of 2 bar is 1350 ppm, using a kagomé-PCF with 15.6 cm length. This value can, however, be much improved by increasing the pressure, the fiber length or the laser power. At 14 bar input pressure, 15.6 cm kagomé-PCF length and 1 W pump power inside the fiber would yield an LOD of approximately 10 ppm for nitrogen using the same setup and an integration time of 1 s.

To demonstrate the potential of this technique the Wobbe index of the natural gas mixture is determined using the relative concentrations of all gas matrix components. Since this is the decisive factor, higher pressures can be used for more accurate readings using the same setup. Using the findings from this paper, the kagomé-PCF is more suitable for this application than the PBG-PCF because of the easier access to spectral region below 1700 cm^−1^. Besides the longer gas exchange time, PBG-PCFs generate a strong glass background, which does not allow facile detection of, e.g., *n*-butane and propane. As these contribute significantly to the heating value of natural gas, accurate determination of the Wobbe index is easier possible in the kagomé-PCF.

**Figure 10:**
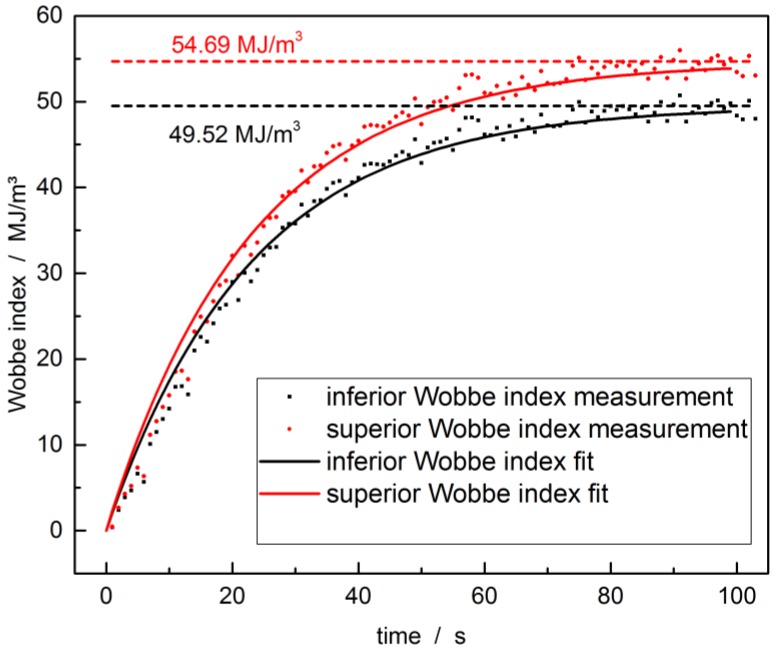
Inferior and superior Wobbe indices during gas exchange in the 80 cm long kagomé-PCF with 2 bar pressure and 28 mW laser power. Dots mark the indices calculated from concentration measurements. The curves are exponential growth fits. The horizontal lines mark the Wobbe indices calculated from the manufacturer specifications for the chosen natural gas mixture. Please note that the venting valve was not employed in this measurement.

Figure 10 shows the Wobbe index obtained from the spectral data taken at a frequency of 1 Hz in a 80 cm long kagomé-PCF at 28 mW laser power and 2 bar input pressure during gas exchange. The purging valve was closed during this measurement, so that about 1 min is needed to reach the equilibrium value in order to highlight the capability of an online determination of the Wobbe index. The speed of Wobbe index determination in a real-world setting is limited by the integration time, the venting, subsequent gas exchange time, and the computing time to determine the intensity of each peak of the Raman spectrum. In conclusion, a Wobbe index determination once every 30 s should be feasible using the currently employed setup and a kagomé-PCF. Assuming that all gas components may be clearly identified a calibration of the reading for the Wobbe index is achieved via the relative strength of individual gas peaks. The measured intensity is therefore set to the known concentration. For comparison, the theoretical Wobbe index is calculated from the gas mixture specifications (horizontal line in Figure 10). The measurement converges well to the theoretical value, which indicates good accuracy of our measurement.

## 4. Conclusions

In this paper we have demonstrated the use of hollow-core PCF to perform fiber enhanced Raman spectroscopy (FERS) in order to enable fast and reliable determination of the Wobbe index of natural gas. To evaluate the suitability in real-world deployments, the influence of fiber length, gas exchange time and input pressure have been determined for two prominent fiber types, namely hollow-core photonic bandgap PCF and kagomé-PCF. While the detected Raman scattering rate of the PBG-PCF is about four times larger, the background signal of the fiber material prevents the easy detection of gaseous molecules with Raman shifts below 1700 cm^−1^, such as *n*-butane and propane. The region around 3000 cm^−1^ could in principle be used to this end but the strong overlap of many hydrocarbons make the signal analysis challenging and prone to inaccuracies. While higher resolution spectrometers for light analysis as well as chemometrics methods may be deployed to resolve this issue, this would incur considerably higher computational and calibration costs. This is the main reason why kagomé-PCF is a better choice for determining the Wobbe index because both *n*-butane and propane feature well-separated rovibrational bands that may be singled out easily in the region below 1700 cm^−1^. Using fibers with even larger core diameter would allow even faster exchange rates. In particular, single-ring fibers [65] could be used, which have very similar properties to kagomé-PCF, also for large core diameters of up to 80 µm. Based on an ideal fiber length, the detection limit of the most prominent gas components has been determined for both types of fiber. The gas exchange times were optimized by purging the dead volume in front of the fiber, allowing exchange of the gas filling of the fiber in a few seconds. The results may pave the way for a robust and fast determination of the natural gas quality at low overall cost. Laser power and gas pressure can be adapted on the basis of the LODs given in this manuscript, so as to design custom systems based on the required detection limit. The optomechanical design of this FERS system may be adapted to higher pressures and gas temperatures such that the maximum ratings are governed by the optical fibers, which can withstand pressures up to 100 bar and temperatures of about 600 °C. The techniques employed here make an on-line Wobbe index measurement with a Raman gas system feasible and results are in good agreement with theoretical values. Furthermore, since the kagomé fibers feature low background scattering other gases such as carbon monoxide (CO), oxygen (O_2_), and hydrogen (H_2_), may be detected with the current setup. For gases with a Raman shifts exceeding 3500 cm^−1^, such as water (H_2_O) at 3652 cm^−1^, the spectrometer has to be recalibrated. However, water adsorption on the inner glass surface of the fiber has been identified as a possible source for increased optical losses in hollow fibers, which is why long term stable operation should prevent the exposure of the fiber to water molecules.

## Figures and Tables

**Table 1:** Composition of the gas mixture used in the experiments according to the manufacturer certificate.

Gas	Specified Molar Concentration [mol %]	Measurement Uncertainty [mol %]
*n*-Butane	0.985	0.020
Propane	3.374	0.067
Ethane	9.50	0.190
Methane	83.93	0.321
Nitrogen	0.4044	0.0081
Carbon dioxide	1.806	0.036

**Table 2:** List of lower and gross heating values as well as the gaseous density of the natural gas components [67,68,69,70].

Gas	Lower Heating Value $H_{i}$ [MJ/m^3^]	Gross Heating Value $H_{s}$ [MJ/m^3^]	Density *ρ* [kg/m^3^]
*n*-Butane	122.910	133.119	2.71
Propane	93.215	101.242	2.01
Ethane	64.345	70.293	1.36
Methane	35.883	39.819	0.72
Nitrogen	0	0	1.25
Carbon dioxide	0	0	1.98

**Table 3:** Theoretical and measured pressure-dependent gas velocities.

Fiber Length/cm	*G_v_*/m s^−1^ bar^−1^	*G_v,theo_*/m s^−1^ bar^−1^
15	(8.0 ± 1.0) × 10^−3^	12.05 × 10^−3^
27	(8.6 ± 0.4) × 10^−3^	6.69 × 10^−3^

**Table 4:** Fit parameters for both types of fibers as well as the offset value for 0 m fiber length.

Fiber Type	$J_{0}$/($\alpha_{S}+\alpha_{P}$)/Hz	$\alpha_{S}+\alpha_{P}$/m^−1^
kagomé	5535 ± 764	4.41 ± 0.69
PBG	29,587 ± 5693	4.94 ± 1.25

**Table 5:** Limit of detection in ppm for optimized fiber lengths and 80 cm long fibers and 1 s integration time at 2 bar input pressure. Higher laser power leads to a linear decrease in the *LOD*.

Gas	PBG-PCF LOD @ 27 mW Pump Power [ppm]		Kagomé-PCF LOD @ 28 mW Pump Power [ppm]		Wave-Number [cm^−1^]	Relative Raman Scattering Intensity
	14.1 cm Length	80 cm Length	15.6 cm Length	80 cm Length		
*n*-Butane	-	-	4140	730	827	1.9 [75]
Propane	-	-	5950	1050	870	1.6 [37,75]
Ethane	2230	440	6050	1070	993	1.2 [37,75]
Methane	62,900	12,300	75,200	13,300	1535	0.1 [75]
Nitrogen	570	110	1350	240	2331	1 [76,77]
Carbon Dioxide	1630	320	4300	760	1388	1.1 [37,75]

## Supplementary Materials

The following are available online at http://www.mdpi.com/1424-8220/17/12/2714/s1, Figure S1: Full spectra of pure nitrogen for the kagomé-type and PBG fiber. Fiber length is 80 cm, applied pressure 2 bar and laser power coupled into the fiber 28 mW and 27 mW respectively, Table S1: Wavenumbers and scattering intensity relative to nitrogen of important natural gas components.
