# Supplementary material for: Monitoring the Wobbe Index of Natural Gas Using Fiber-Enhanced Raman Spectroscopy
Source: Sensors (Basel). 2017 Nov 24;17(12):2714. doi: 10.3390/s17122714 (PMC5753068; doi:10.3390/s17122714)
Supplement: Supplementary file 1 [file sensors-17-02714-s001.pdf]

sensors

Supplementary Material

Monitoring the Wobbe Index of Natural Gas Using Fiber-Enhanced Raman Spectroscopy

Vincenz Sandfort [1], Barbara M. Trabold [2], Amir Abdolvand [2,3], Carsten Bolwien [4], Philip St.J. Russell [2], Jürgen Wöllenstein [1,4] and Stefan Palzer [5,*]

1 Laboratory for Gas Sensors, Department of Microsystems Engineering–IMTEK, University of Freiburg, Georges-Köhler-Allee 102, 79110 Freiburg, Germany; vincenz.sandfort@imtek.uni-freiburg.de
2 Max Planck Institute for the Science of Light, Staudtstraße 2, 91058 Erlangen, Germany; barbara.trabold@mpl.mpg.de (B.M.T.); aabdolvand@ntu.edu.sg (A.A.); philip.russell@mpl.mpg.de (P.S.J.R.)
3 School of Electrical and Electronic Engineering, Nanyang Technological University, 50 Nanyang Ave, Singapore 639798, Singapore
4 Fraunhofer Institute for Physical Measurement Techniques IPM, Heidenhofstraße 8, 79110 Freiburg, Germany; Carsten.Bolwien@ipm.fraunhofer.de (C.B.); Juergen.Woellenstein@ipm.fraunhofer.de (J.W.)
5 Department of Computer Science, Universidad Autónoma de Madrid, Francisco Tomás y Valiente 11, 28049 Madrid, Spain
* Correspondence: stefan.palzer@uam.es

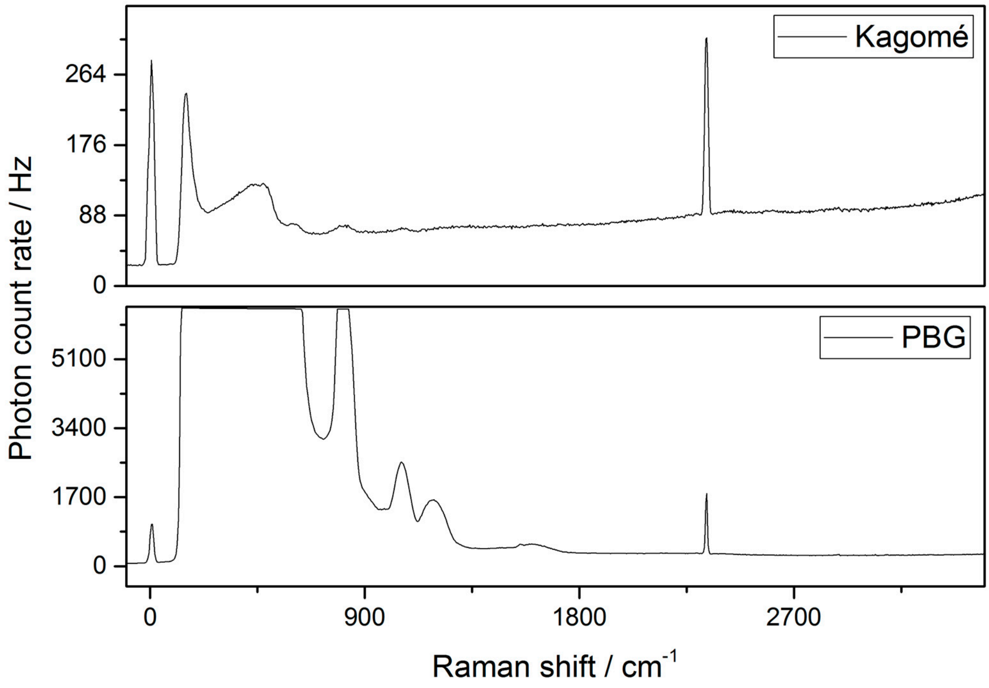

Figure S1. Full spectra of pure nitrogen for the kagomé-type and PBG fiber. Fiber length is 80 cm, applied pressure 2 bar and laser power coupled into the fiber 28 mW and 27 mW respectively.

Table S1. Wavenumbers and scattering intensity relative to nitrogen of important natural gas components.

Wavenumber [cm^{-1}]	Gas	Relative intensity
827	*n*-Butane	1.9 [1]
870	Propane	1.6 [1,2]
993	Ethane	1.2 [1,2]
1286	Carbon dioxide	0.8 [1,2]
1388	Carbon dioxide	1.1 [1,2]

1535	Methane	0.1 [1]
2331	Nitrogen	1 [3,4]
2890	*n*-Butane	15.6 [1]
2908	Propane	19.6 [1,2]
2914	Ethane	15.0 [1,2]
2917	Methane	8.6 [1,2]

References

1. Kiefer, J.; Seeger, T.; Steuer, S.; Schorsch, S.; Weikl, M. C.; Leipertz, A. Design and characterization of a Raman-scattering-based sensor system for temporally resolved gas analysis and its application in a gas turbine power plant. *Meas. Sci. Technol.* **2008**, *19*, 85408, doi:10.1088/0957-0233/19/8/085408.
2. Bougeard, D.; Buback, M.; Cao, A.; Gerwert, K.; Heise, H. M.; Hoffmann, G. G.; Jordanov, B.; Kiefer, W.; Korte, E.-H.; Kuzmany, H.; Leipertz, A.; Lentz, E.; Liquier, J.; Röseler, A.; Schnöckel, H.; Schrader, B.; Schrötter, H. W.; Spiekermann, M.; Taillandier, E.; Willner, H. *Infrared and Raman spectroscopy: methods and applications*; Schrader, B., Ed.; Wiley-VCH Verlag GmbH: Weinheim, Germany, 2008; ISBN 3527615423.
3. Eichmann, S. C.; Weschta, M.; Kiefer, J.; Seeger, T.; Leipertz, A. Characterization of a fast gas analyzer based on Raman scattering for the analysis of synthesis gas. *Rev. Sci. Instrum.* **2010**, *81*, 125104.
4. Fenner, W. R.; Hyatt, H. a; Kellam, J. M.; Porto, S. P. S. Raman cross-section of some simple gases. *J. Opt. Soc. Am.* **1973**, *63*, 73–77, doi:10.1364/JOSA.63.000073.
